# Enhanced clinical-scale manufacturing of TCR transduced T-cells using closed culture system modules

**DOI:** 10.1186/s12967-018-1384-z

**Published:** 2018-01-24

**Authors:** Jianjian Jin, Nikolaos Gkitsas, Vicki S. Fellowes, Jiaqiang Ren, Steven A. Feldman, Christian S. Hinrichs, David F. Stroncek, Steven L. Highfill

**Affiliations:** 1 Center for Cellular Engineering, Department of Transfusion Medicine, Clinical Center, National Institutes of Health, 10 Center Drive, MSC-1184, Building 10, Room 3C720, Bethesda, MD 20892-1184 USA; 2 Experimental Transplantation and Immunology Branch, National Cancer Institute, NIH, Bethesda, MD USA; 3 Surgery Branch, National Cancer Institute, NIH, Bethesda, MD USA

**Keywords:** E6 HPV, E7 HPV, HPV-16+, T-cell receptor, Cellular therapy, Cancer immunotherapy, Cervical cancer, Epithelial cancer, T-cell manufacturing

## Abstract

**Background:**

Genetic engineering of T-cells to express specific T cell receptors (TCR) has emerged as a novel strategy to treat various malignancies. More widespread utilization of these types of therapies has been somewhat constrained by the lack of closed culture processes capable of expanding sufficient numbers of T-cells for clinical application. Here, we evaluate a process for robust clinical grade manufacturing of TCR gene engineered T-cells.

**Methods:**

TCRs that target human papillomavirus E6 and E7 were independently tested. A 21 day process was divided into a transduction phase (7 days) and a rapid expansion phase (14 days). This process was evaluated using two healthy donor samples and four samples obtained from patients with epithelial cancers.

**Results:**

The process resulted in ~ 2000-fold increase in viable nucleated cells and high transduction efficiencies (64–92%). At the end of culture, functional assays demonstrated that these cells were potent and specific in their ability to kill tumor cells bearing target and secrete large quantities of interferon and tumor necrosis factor. Both phases of culture were contained within closed or semi-closed modules, which include automated density gradient separation and cell culture bags for the first phase and closed GREX culture devices and wash/concentrate systems for the second phase.

**Conclusion:**

Large-scale manufacturing using modular systems and semi-automated devices resulted in highly functional clinical-grade TCR transduced T-cells. This process is now in use in actively accruing clinical trials and the NIH Clinical Center and can be utilized at other cell therapy manufacturing sites that wish to scale-up and optimize their processing using closed systems.

## Background

Cellular immunotherapy for the treatment of cancer has undergone remarkable growth over the past few years. Much of the recent attention that has led to this increased development has been due to encouraging phase I/II clinical trials using CAR T-cells directed against CD19 and CD22 [1–3] and TCR-modified T-cells directed against NY-ESO-1, MAGE, and GP100 [4–7]. Both of these approaches have their own particular nuances. While TCR-transduced T-cells are able to recognize intracellular antigens processed by major histocompatibility (MHC) proteins, CAR T-cells only target surface antigens, but these can be recognized in a MHC-independent fashion, allowing more patients to be treated with a specific viral vector.

The human papillomavirus (HPV) E6 and E7 oncoproteins are required for the induction and maintenance of the malignant phenotype of HPV-associated cancers. These oncoproteins have been postulated to be ideal targets in patients with HPV-associated epithelial cancers in that they are constitutively expressed by HPV cancers and not expressed in healthy tissue [8]. Currently, three separate vaccines for HPV-16 are approved for clinical use and have demonstrated excellent safety profiles (Gardasil^®^, Gardasil^®^9, and Cevarix^®^). While these vaccines are highly effective in preventing new HPV infections, they are incapable of treating established HPV infections [9, 10].

A method was developed to expand E6/E7-specific tumor infiltrating lymphocytes (TIL) to treat patients with HPV-positive metastatic cervical cancer [11, 12]. In a recent clinical trial (NCT01585428), three out of nine patients experienced objective tumor responses, two of which were durable complete responses [13]. Interestingly, it was later discovered that the two patients that developed a complete response (CR) not only had viral-specific T-cells present in their blood, but also, harbored T-cells with reactivity against mutated neoantigens [14]. The positive outcome in this trial led to efforts to target HPV-positive cancers with T-cells that are genetically engineered with TCRs against HPV-16 E6 and E7 [15]. Preclinical studies demonstrated that these engineered T-cells had high avidity, effector cytokine production and lytic ability against target cell lines, which paved the way for three separate clinical trials at the National Cancer Institute (NCI-NCT02280811, NCT03197025, NCT02858310).

A major limiting factor for the administration of TCR-transduced T-cells is the seemingly large number of cells required to obtain a clinical response. For example, the average number of T-cells infused in the 6 of the 20 patients that experienced a partial tumor response using T-cells transduced with a MART1-specific TCR was 21.5 × 10^9^ cells (range of 3.8–48 × 10^9^ cells) [6]. In another study utilizing NY-ESO-1-specific TCR transduced T-cells, the average cell dose that achieved at least a partial response was 66.4 × 10^9^ cells for patients with melanoma (range of 37–130 × 10^9^ cells; n = 5/11) and 62 × 10^9^ cells for patients with synovial cell carcinoma (range of 50–83 × 10^9^ cells; n = 4/6) [16]. Manufacturing procedures that were used to generate the large number of cells needed in these trials utilized 6-well plates for transduction and culture flasks for expansion, which puts the product at higher risk for microbial contamination. It is notable that we and other cell therapy manufacturing centers are moving more toward automated and closed system approaches to manufacture CAR T-cells in order to improve consistency and avoid potential microbial contamination [17]. Unfortunately, the current yield of these fully automated and closed single systems ranges from ~ 1–5 × 10^9^ cells, which is sufficient to treat patients enrolled on most CAR T-cell protocols for hematologic malignancies, but insufficient for many TCR T-cell protocols which can require up to 100 × 10^9^ total cells. Here, we investigated the possibility of manufacturing large numbers of transduced T-cells in closed and semi-closed, modular systems with the hope that this will result in a more safe and reliable manufacturing procedure. This procedure was designed and performed using TCR-transduced T-cells, but is easily adapted for CAR T-cells and other T-cell cultures that may require viral transduction, making it an important advancement in GMP manufacturing of cellular immunotherapy.

## Methods

### Study participants

The patient samples studied were acquired with informed consent from patients enrolled on National Cancer Institute (NCI)(Bethesda, MD) Protocol Number 03-C-0277, which was approved by the NCI Institutional Review Board. Leukapheresis was performed by the Surgery Branch Leukapheresis Unit. Peripheral blood mononuclear cells (PBMC) were isolated from leukapheresis samples and cryopreserved by the Surgery Branch TIL Laboratory using standard operating procedures. Healthy donor samples were collected after obtaining informed consent by the Department of Transfusion Medicine, Clinical Center, NIH on protocol 99-CC-0168.

### Construction and production of clinical-grade E6 and E7 TCR recombinant retroviral vector

The PG13-MSVG1-E7 TCR (B3) retroviral vector and PG13-MSGV1-oDCA2-E6 TCR retroviral vector was prepared and cryopreserved following cGMP conditions in the Surgery Branch Vector Production Facility (SBVPF), NCI, NIH.

### Preparation of peripheral blood mononuclear cells

Healthy donor volunteers and patients who were enrolled into clinical protocols signed informed consent that were approved by the Institutional Review Board of the National Institute of Health. Peripheral Blood Mononuclear Cells (PBMCs) were collected by leukapheresis and were enriched for lymphocytes by automated ficoll density gradient separation using COBE2991 Cell Processor (TERUMO, Lakewood, CO). Following separation, PBMCs were either immediately cultured for transduction or cryopreserved for later culture/transduction. For feeder cells used in the Rapid Expansion Protocol (REP), PBMCs from healthy donors were cryopreserved after density gradient separation.

### Generation of E6 and E7 TCR transduced T-cells

Fresh or frozen and thawed PBMCs were cultured with TCR-300 CM which contains AIM V medium (Gibco, Grand Island, NY), 5% heat-inactivated human AB Serum (Valley Biomedical, Winchester, VA), 2 mM GlutaMax (Gibco), and 300 IU/mL IL-2 (Prometheus Laboratory, Inc. San Diego, CA). The cells were stimulated with 50 ng/mL soluble anti-CD3 (Miltenyi Biotec, Auburn, CA) and placed in flasks for 48 h at 37°, 5% CO_2_. On day 2 post activation, the cells were transduced with E6 TCR or E7 TCR retroviral vectors by spinoculation and overnight incubation in a closed bag system. PermaLife bags (OriGen Biomedical, Austin, TX) were coated with RetroNectin (Takara Bio, Mountain View CA) overnight. The bags were then incubated with a blocking solution containing PBS with 2.5% HSA, and rinsed with a wash solution (WS) containing HBSS and 25 mM HEPES. Retroviral vector (15 mL) was added into each bag, and centrifuged at 2000*g* for 2 h at 32 °C. Viable cells (15 × 10^6^) were added into each bag to a final concentration of 0.5 × 10^6^/mL, and the bags were centrifuged at 1000*g* for 15 min at 32 °C. The bags containing the cell and viral suspension were placed in a 37 °C incubator overnight. The procedure was repeated on day 3 for the 2nd transduction. On day 4, the transduction was stopped and cells were diluted to 0.4 × 10^6^ cells/mL with fresh TCR-300 CM. Cell were expanded until day 7–10. The transduced cells were harvested and cryopreserved or initiated fresh in the REP.

### Rapid expansion protocol (REP) for transduced cells

REP was initiated with fresh or cryopreserved transduced cells. The transduced cells were cultured with irradiated (50 Gy) allogeneic PBMCs from three healthy donors as “feeder” cells at a ratio of 1 to 100. The cultures were initiated in closed, gas-permeable G-REX500MCS vessel (Wilson Wolf Manufacturing, New Brighton, MN). For each G-REX500MCS, 10 × 10^6^ viable cells and 1 × 10^9^ irradiated feeders were cultured in 800 mL of REP-3000-5 CM containing AIM-V medium, 2 mM GlutaMax, 3000 IU/mL IL-2 and 5% heat-inactivated human AB Serum, and supplemented with 30 ng/mL of anti-CD3. The vessels were incubated at 37 °C in 5% CO_2_. Four days after culture initiation, 800 mL of REP-3000-5 CM was added to each vessel to a final volume of 1600 mL. On day 7, additional 1200 mL of REP-3000-5 CM was added to each vessel. On day 11, REP-3000-0 CM was prepared, which contains AIM-V medium, 2 mM GlutaMax, and 3000 IU/mL IL-2. Thousand seven hundred milliliter of REP-3000-0 CM was added to each flask to a final volume of 4500 mL. The cells were harvested on day 14 of culture. At harvest, the supernatant of each G-REX500MCS vessel was removed by GatherREX (Wilson Wolf Manufacturing) to reduce volume of cell suspension for concentration and wash. The cell suspension was then concentrated and washed using the LOVO device (Fresenius Kabi, Lake Zurich, IL). The wash solution is plasmalyte-A (Baxter, Deerfield, IL) supplemented with 0.5% HSA (Baxter). After the washing procedure was complete, the cell product was supplemented with 4% HSA in plasmalyte-A.

### Cell counts and flow cytometry

Cell counts were performed using the Advia 120 automated hematology analyzer (Siemens Healthcare, Erlangen, Germany) and Cellometer Auto 2000 (Nexcelom Bioscience, Lawrence, MA). Flow cytometry was performed with a FACSCanto II (BD Biosciences, San Jose, CA) using CD3, CD4, CD8, CD14, CD15, CD19, CD45 and CD56 antibodies (BD Biosciences). The expression of E6 TCR and E7 TCR was assessed by flow cytometry using antibodies that recognize murine components within the TCR construct (anti-mouse TCRβ).

### Cytotoxicity assays

Killing activity was determined using the xCELLigence RTCA MP (Acea Bioscineces Inc., San Diego, CA) instrument and was calculated by measuring electrical impedance in the culture plates caused by the adhering target cell lines. Addition of the non-adhering TCR cells at a ratio of 1:1 (E:T) results in decreasing electrical impedance measured in the culture wells due to cell death and cytolysis of the target cells. Cytolytic activity was measured in percentage against wells that contain either only target cells or effector cells. Electrical impedance was calculated every 15 min.

### Cytokine secretion assays

E6 or E7 TCR transduced T-cells were co-cultured with the target cell lines at a ratio of 1:1 for 24 h in 96-well plates. The plates were centrifuged and the supernatants removed and stored in − 30 °C. ELISA kit’s were purchased from RnD Systems (Minneapolis, MN) and were used according to manufacturer’s instructions. Briefly, Assay Diluent was mixed with supernatant sample (diluted 1:10 in advance) and incubated in room temperature for 2 h. Consecutively, the microplates were washed 4× and either TNF-α or IFN-γ conjugates were added and incubated for further 2 h. The microplates were washed and Substrate solution was added to the microplates and incubated for about 20 min in room temperature protected from light. Stop solution was added to each well to stop the reaction. On the same plates and for each assay, a standard curve was created according to manufacturer’s instructions from the provided Stock solution of known concentration. The microplates were read for Optical Density using an AccuSKAN FC Microplate photometer (Thermo Fisher Waltham, MA) at 450 nm with a wavelength correction at 540 nm. The optical density results were analyzed and plotted using Prism. All optical densities were corrected against the optical densities of control wells on the microplates. The standard curves created (with R^2^ values of 0.9911 and 0.9964 for IFN-γ and TNF-α respectively) were used to calculate the optical densities to actual concentrations. All samples were done in triplicate.

### Graphs and statistical analysis

Graphs were generated using Graphpad Prism 7 Software. For cytotoxicity assays and cytokine secretion assays, *P* values were calculated using Students *t* test for comparing like groups. *P* < 0.05 was considered statistically significant and is illustrated with an asterisk (*).

## Results

### Viability and expansion characteristics following cell activation and transduction

**Fig. 1:**
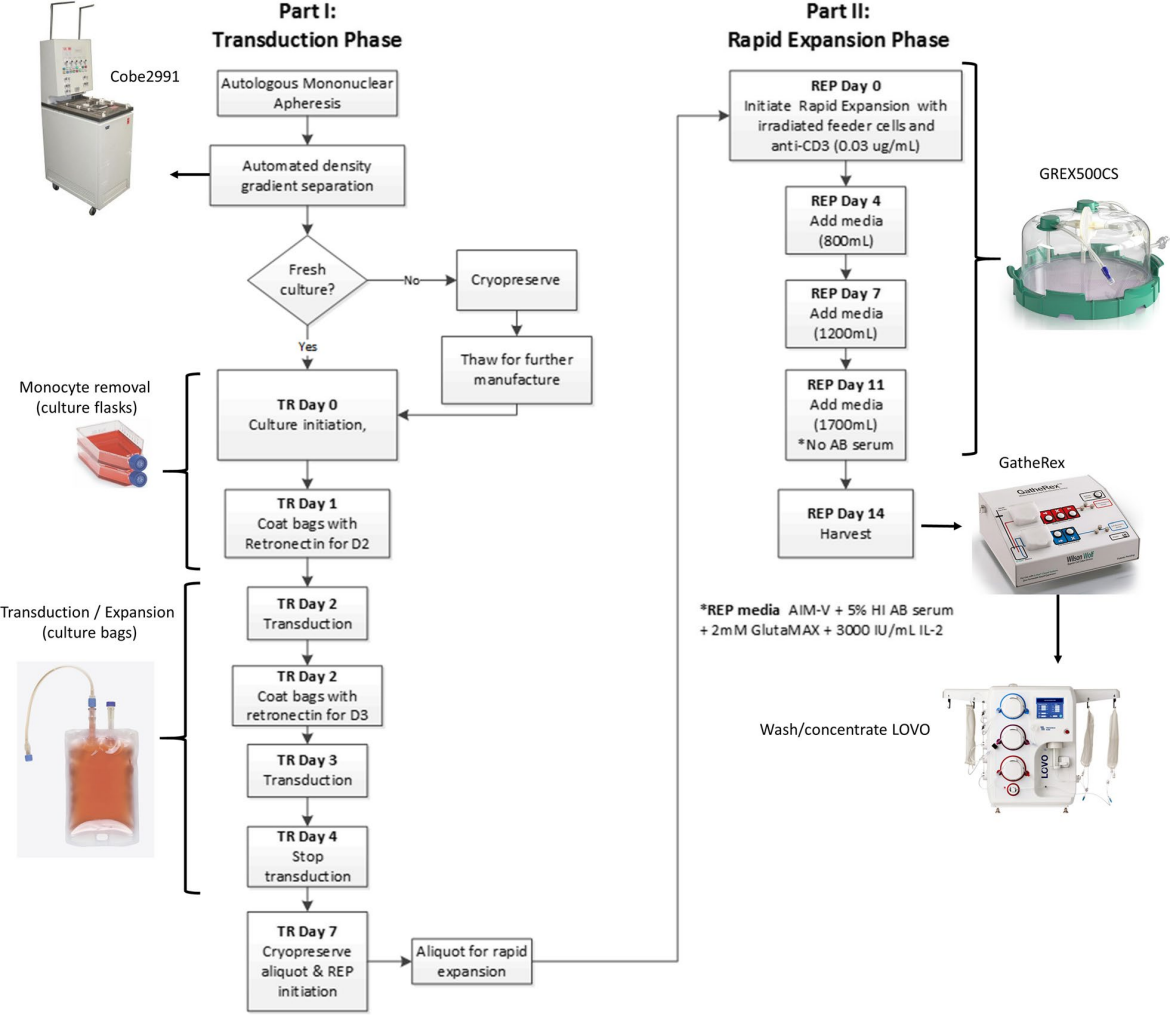
Manufacturing schema. The manufacturing schema for E6 TCR- and E7 TCR-specific T-cells is shown. Part I (*left*) outlines the transduction phase of the culture and extends to day 7. At the end of this phase of culture, cells can be cryopreserved or a fresh initiation can occur for Part II, the rapid expansion phase of the culture (*right*). Here, TCR-transduced T-cells are mixed with allogeneic feeder cells from three different donors and allowed to expand in closed G-REX500 culture devices for a period of 14 days

We developed a new manufacturing procedure for the expansion and transduction of human T-cells with gamma-retroviral vectors using closed and semi-closed modular systems (Fig. 1). Part 1 of our manufacturing process consisted of an activation and transduction phase that lasted 7 days. Here, mononuclear apheresis was obtained from healthy donors or patients with HPV-16+ epithelial cancer. Lymphocytes were enriched in a closed process through density gradient separation using Ficoll and cells were activated with anti-CD3 (OKT3; 50 ng/mL) in cell culture flasks for 2 days. Culture flasks were utilized to aid in the removal of tumor-induced monocytes, which are known to inhibit T-cell activation and expansion [18, 19].

**Fig. 2:**
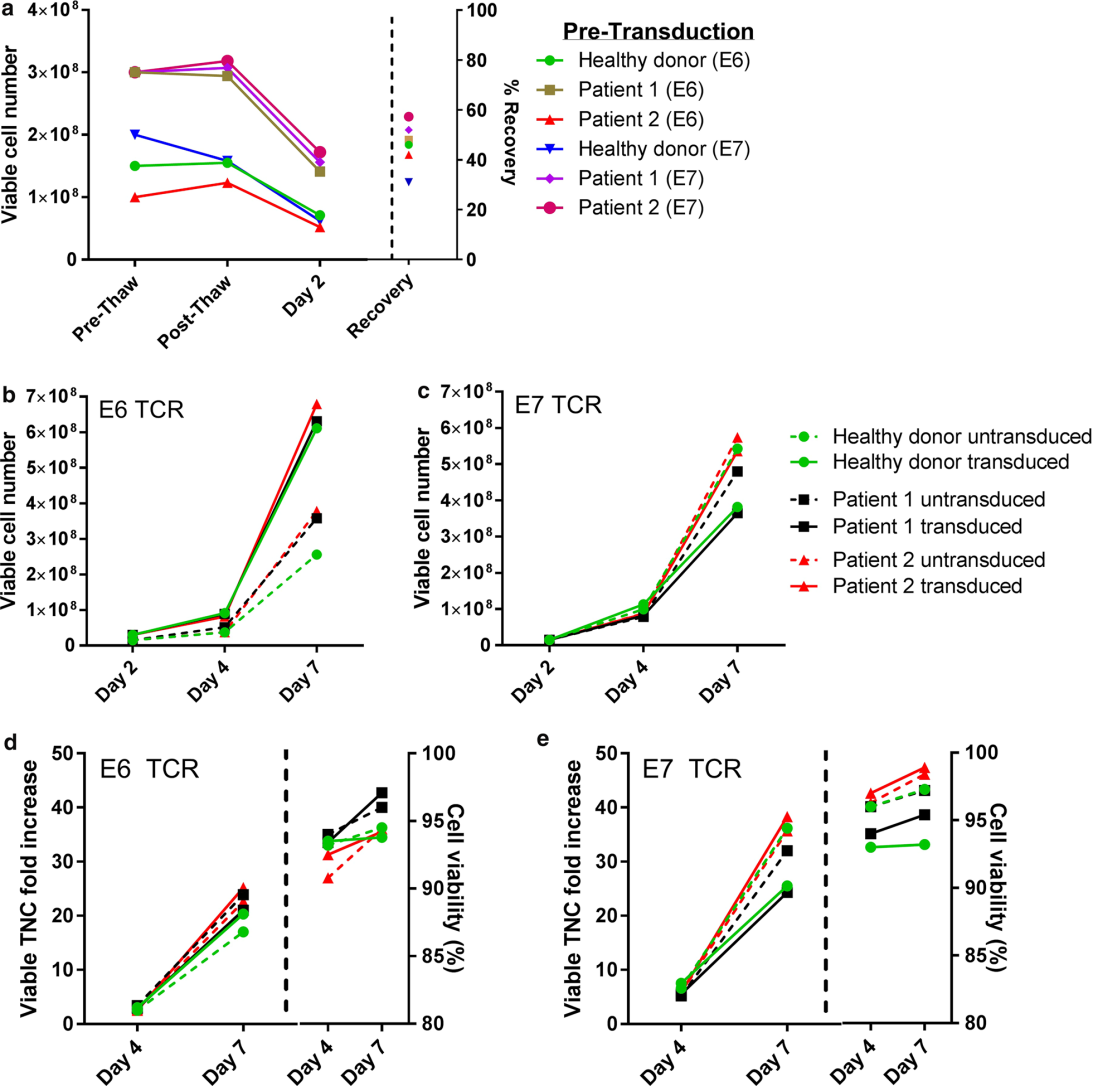
Viability and expansion characteristics following cell activation and transduction. **a** Cell viability and absolute numbers were measured during the initiation of phase I of culture, percent recovery of cells from the pre-thaw cryopreserved aliquot was measured at day 2 of culture. Viable cell number for E6 TCR (**b**) and E7 TCR (**c**) cultures were plotted at the days indicated. Viability was monitored and Fold increase at day 4 and 7 was calculated from the day 2 starting point for E6 TCR (**d**) and E7 TCR (**e**) cultures

On day 2 and day 3 following activation, culture bags were sterilely welded and transferred to closed RetroNectin-coated bags and transduced with retroviral vector. Cells were washed at day 4 and allowed to expand within the bags until day 7. The absolute number of viable cells was determined prior to cryopreservation, after thaw, and on day 2 of culture prior to T-cell transduction. No significant drop in cell number was observed during the thaw process, however, cell number decreased significantly from post-thaw to day 2 of culture. This drop in viable cell number was due to cell death and monocyte removal (Fig. 2a). A consistent pattern was observed among the six samples tested regardless of whether samples were from healthy donors or patients, or when differing numbers of cells were used at cryopreservation.

Viable cell number was monitored from day 2 post transduction through day 7 of culture and transduced T-cells expanded from 15 × 10^6^ to 6.4 × 10^8^ ± 3.5 × 10^7^ cells for E6 cultures and 4.3 × 10^8^ ± 9.4 × 10^7^ cells for E7 cultures, with most of the cell expansion occurring between day 4 and day 7 of culture (Fig. 2b, c). Viable fold increase for this transduction phase for E6 TCR transduced T-cells at day 7 was 21.3-fold ± 1.2 and for E7 TCR transduced T-cells was 28.5-fold ± 6.2 (Fig. 2d, e). Viability during this portion of culture remained > 93% for all samples tested (Fig. 2d, e right panel). In summary, healthy donor samples and patient samples for both E6 and E7 TCR transduced T-cells had a very consistent and robust expansion with high viability at the end of phase 1 of culture.

### T-cells are transduced and selectively expanded at high efficiency following the activation phase of culture

**Fig. 3:**
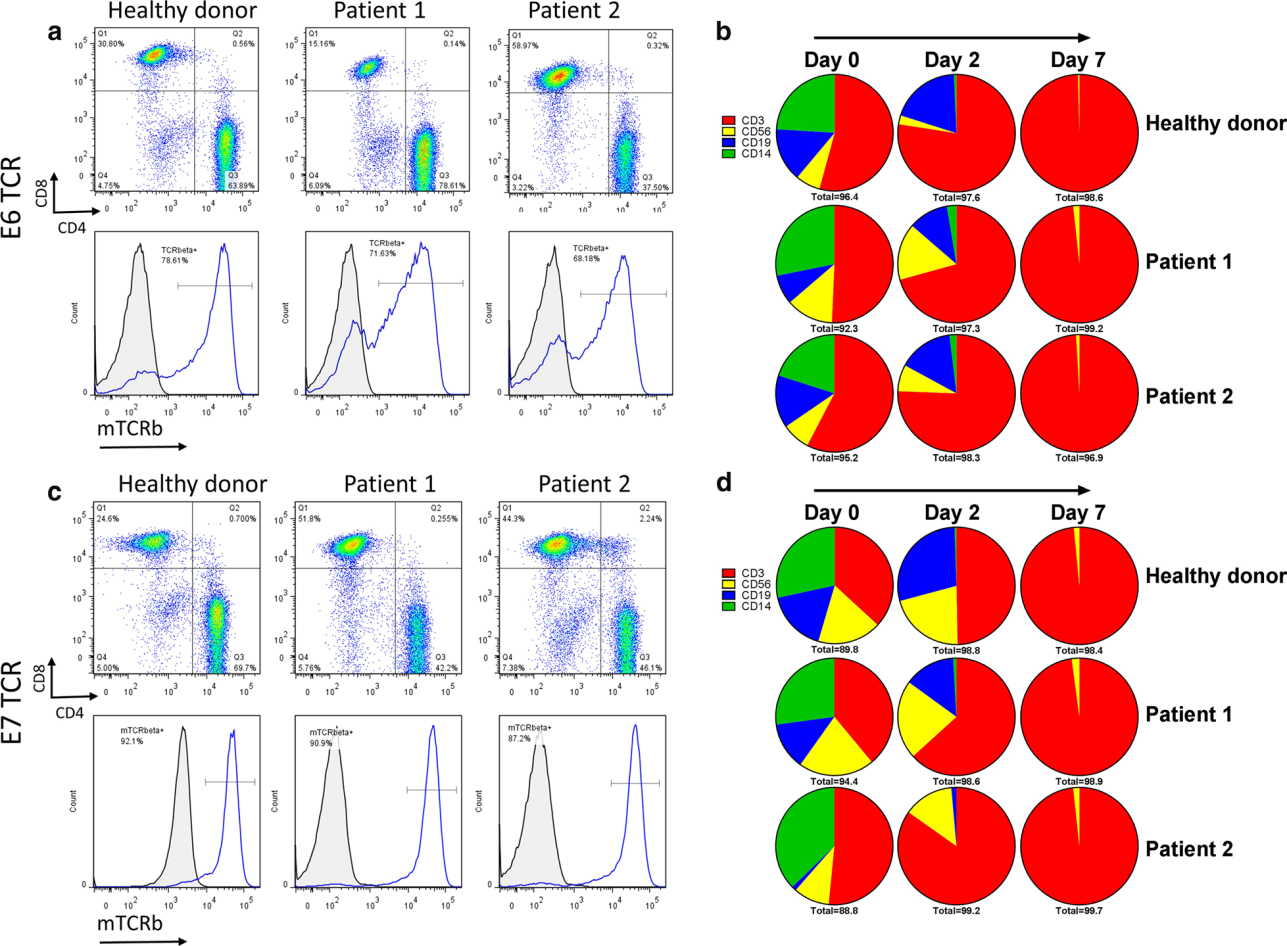
T-cells are transduced and selectively expanded at high efficiency following the activation phase of the culture. Samples were taken at day 7 of culture and flow cytometric analysis was performed to determine the phenotype of the culture and the transduction efficiency of the T-cells. FACS plots of (**a**) E6 TCR- and (**c**) E7 TCR-transduced cultures show CD4 and CD8 ratios (*top*) and transduction efficiencies of CD3-gated T-cells (*bottom*). **b**, **d** The ratios of T-cells (CD3), NK cells (CD56), B-cells (CD19) and monocytes (CD14) were monitored throughout day 7 of phase I of the culture

Flow cytometry was used to monitor transduction efficiency and changes in cell populations within the culture over time. The CD4/CD8 ratio between healthy donor and patient samples was variable at day 7, with the healthy donor samples from both E6 and E7 TCR and patient 1 sample from E6 TCR favoring CD4 T-cells. (Fig. 3a, c). Transduction efficiency was highly consistent for each group with E6 TCR transduced cells having a transduction efficiency of 73% ± 5.3 and E7 TCR transduced cells having 90% ± 2.6 (Fig. 3a, c). Cell populations in the culture were also quite variable at day 0 among all of the samples tested, but despite these early differences, all cultures were > 96% CD3+ T-cells by day 7 of culture (Fig. 3b, d). The high efficiency of monocyte removal between day 0 and day 2 was due to the culture initiation in cell culture flasks.

### Rapid expansion of T-cells is robust within a closed system

**Fig. 4:**
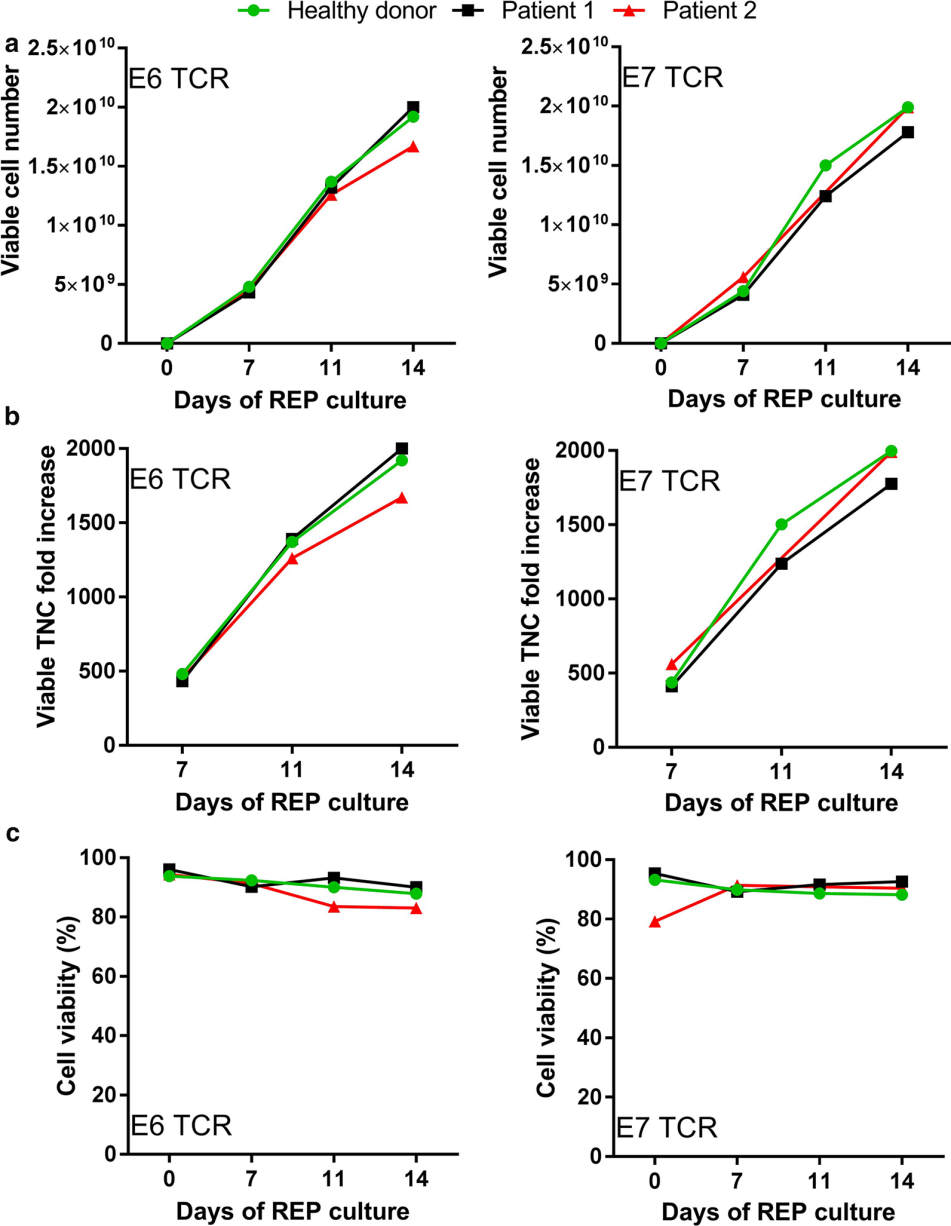
Robust rapid expansion in a closed manufacturing system. Samples were removed from culture and absolute viable cell number (**a**), viable TNC fold increase (**b**), and cell viability (**c**) are reported for days 7, 11, and 14 of REP. E6 TCR transduced T-cells (*left*) and E7 TCR transduced T-cells (*right*)

Part two of the manufacturing process was devoted to rapidly expanding the cells to achieve the numbers needed for clinical administration (REP, rapid expansion protocol). Ten million viable T-cells were removed from cell culture bags and placed in a closed G-REX500CS container with irradiated feeder cells isolated from three different allogeneic donors plus anti-CD3 (OKT3; 30 ng/mL) and 3000 IU/mL IL-2. Additional media was added to supplement growth of expanding T-cells at day 4 (11), 7 (14) and 11 (18) of REP culture and cells were harvested at day 14 (21) after REP culture initiation (total culture days). Viable cell number was monitored at day 7, 11, and 14 of REP and was highly consistent within the E6 TCR and E7 TCR transduced groups, and also between the two groups. We were able to generate approximately 18.9 × 10^9^ ± 1.4 × 10^9^ T-cells by the end of the culture for all samples (Fig. 4a). In accordance with these results, viable TNC fold increase for the REP portion of culture was also very consistent and was 1890 ± 138-fold increase for all samples tested (Fig. 4b), and cell viability was greater than 83% in the final product (Fig. 4c). Therefore, expansion and viability of cells within this closed culture apparatus proved to be favorable for producing a sufficient quantity of T-cells for clinical use.

### Transduction efficiency remains high after rapid expansion

**Fig. 5:**
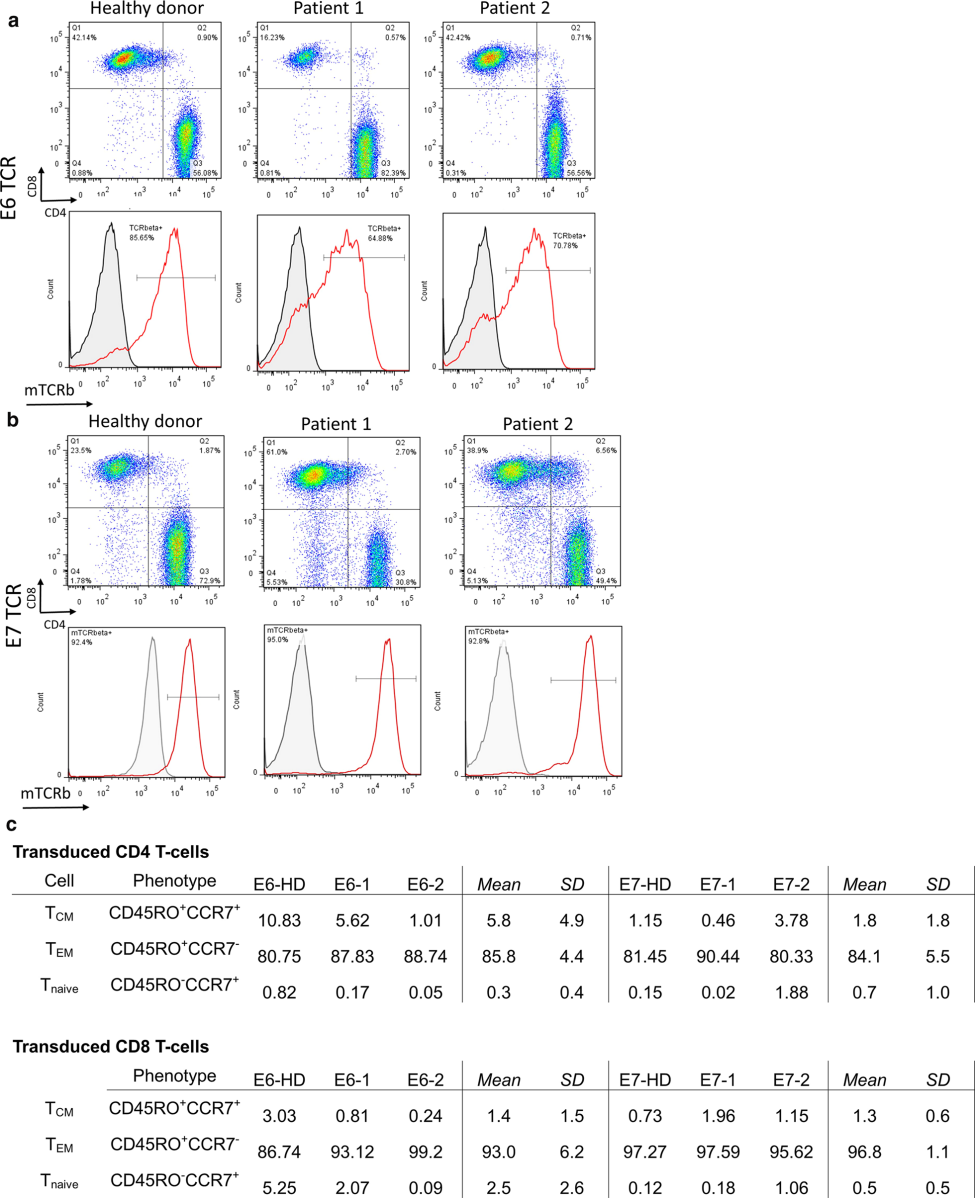
Transduction efficiency and cell phenotype following Rapid Expansion. Samples were taken at day 14 of REP culture and flow cytometric analysis was performed to determine the phenotype of the culture and the transduction efficiency of the T-cells. FACS plots of (**a**) E6 TCR- and (**b**) E7 TCR-transduced cultures show CD4 and CD8 ratios (*top*) and transduction efficiencies of CD3-gated T-cells (*bottom*). **c** Cell phenotype for CD4 and CD8 transduced cells is further characterized as T_CM_ (CD45RO^+^CCR7^+^), T_EM_ (CD45RO^+^CCR7^−^), or T_naive_ (CD45RO^−^CCR7^+^). Mean and SD between cultures are shown

Next, we ascertained the transduction efficiency and CD4/CD8 T-cell ratio of the final product following Rapid Expansion. CD4+ T-cells were favored in all samples except one (Patient 1 of E7 TCR), and final transduction efficiency of E6 TCR transduced T-cells was 74 ± 10% and E7 TCR transduced T-cells was 93 ± 1.4% (Fig. 5a, b). We also examined CD4 and CD8 T-cell phenotype by flow cytometry for the expression of markers associated with naïve, effector memory, or central memory phenotype (T_naive_, T_EM_, T_CM_). As is typically seen, T_EM_ cells represented the dominant fraction within the final product (Fig. 5c).

### E6 and E7 TCR transduced T-cells are highly lytic and secrete large quantities of pro-inflammatory cytokines when combined with cell lines expressing specific target

**Fig. 6:**
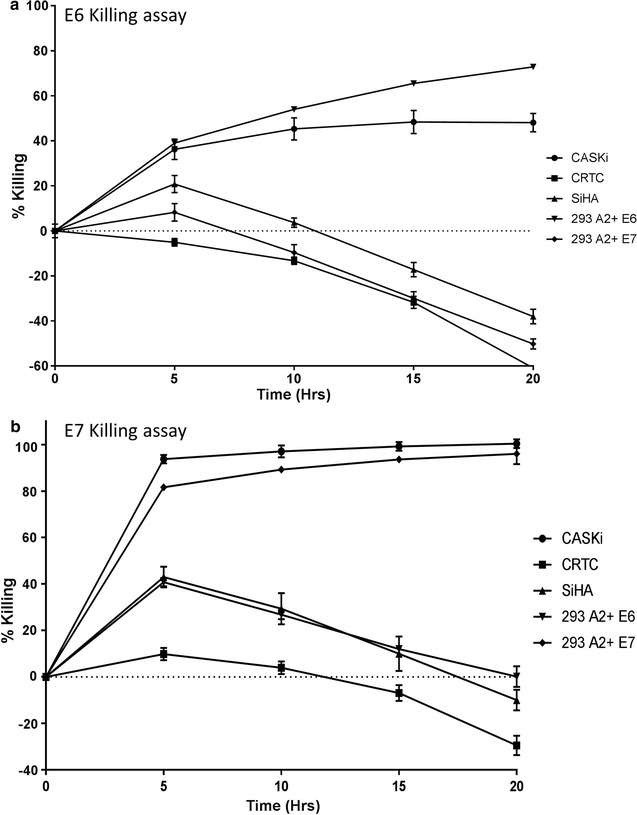
E6/E7 TCR transduced T-cells have high specific lytic activity against target cell lines. Representative comparison of cytolytic activity of the E6 (**a**) and E7 (**b**) TCR transduced cells against five target cell lines. Averages and SD of triplicate wells are shown. Target cells lines used are: E6-expressing cell line (CASKi), positive control E6+ cell line (293-E6+), E6/E7 negative control cell line (CRTC), HLA-A2 negative cell line (SiHA) and E6–E7+ control cell line (293-E7 +)

The ability of E6 TCR and E7 TCR transduced T-cells to identify and kill target cell lines expressing HPV-16 antigen was evaluated next. Here, 1 × 10^3^ transduced T-cells were mixed with 1 × 10^3^ target cells naturally expressing target antigen (CASKi, E6 and E7) or 293 cells stably transduced to express target antigen in the context of HLA-A2 and incubated at 37 °C for up to 20 h. E6 TCR transduced T-cells were able to specifically lyse CASKi cells (48 ± 4%) and 293 cells expressing E6 (72 ± 1%) at the 20 h time point (Fig. 6a). E7 TCR transduced T-cells possessed higher lytic ability and killed 100 ± 1.9% CASKi cells and 96 ± 4% 293 cells expressing E7 target at the 5 h time point (Fig. 6b). Importantly, cell lines not expressing the target antigens E6/E7, but expressing HLA-A2 (CRTC, SiHA) had significantly diminished cell death at all time points and were proliferating in culture at the 20 h time point. Also, specificity of target antigen lysis was high given that E6 TCR transduced T-cells were unable to recognize cells transduced with E7+ HLA-A2 and vice versa.

**Fig. 7:**
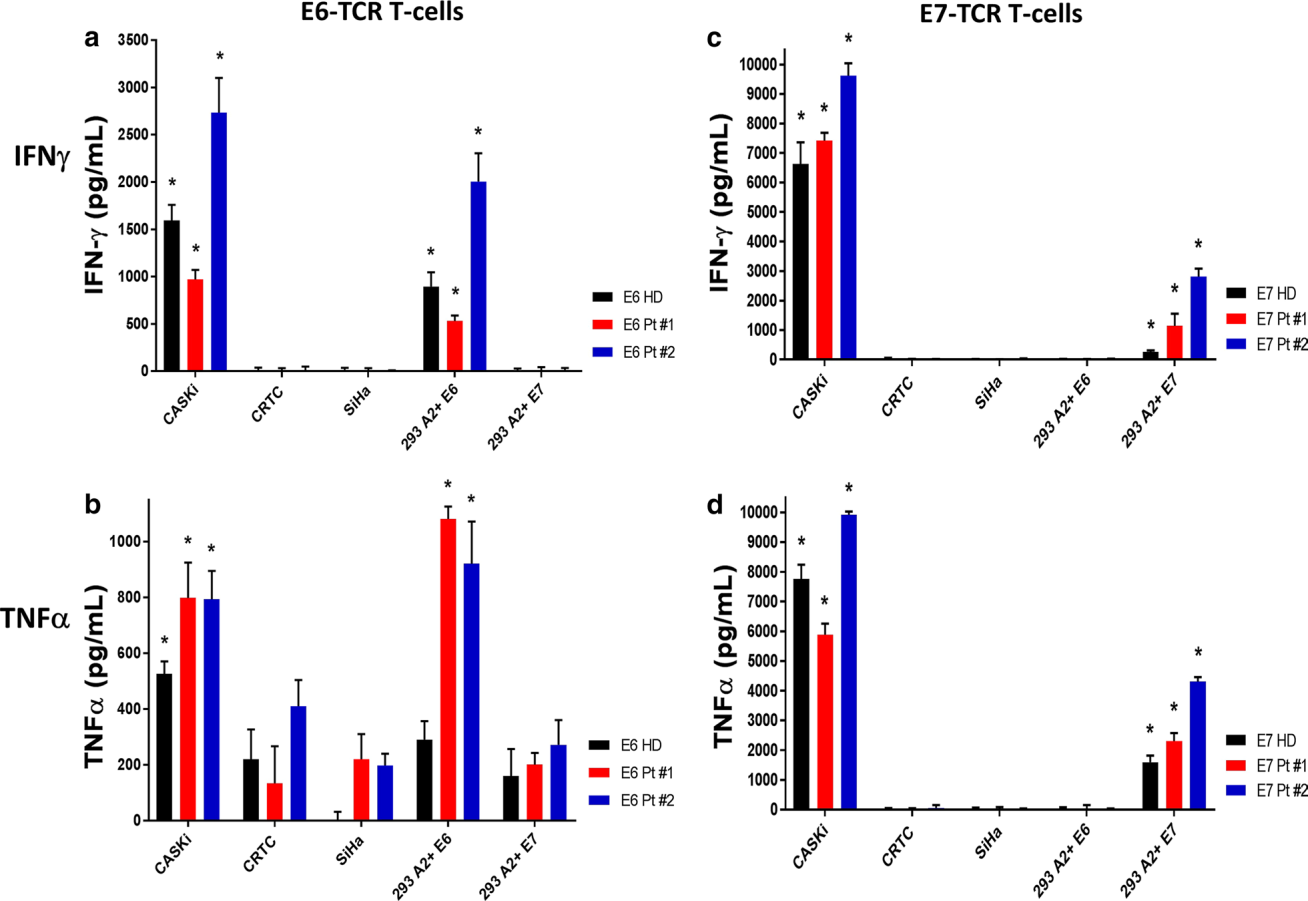
E6/E7 TCR transduced T-cells secrete large quantities of pro-inflammatory cytokines when co-cultured with cell lines expressing specific target. E6 (**a**, **b**) and E7 (**c**, **d**) TCR T cells were co-cultured overnight with five target cell lines at a ratio of 1:1. Culture supernatants were analyzed for IFN-γ (top row) and TNF-α (bottom row). Mean and SD of triplicate wells are shown. * Indicates p < 0.05 between selected group versus all negative control groups

Finally, we examined the ability of the TCR transduced T-cells to generate pro-inflammatory cytokines upon recognition of target antigen. E6 and E7 TCR transduced T-cells produced significant quantities of IFN-γ and TNF-α in a target-dependent manner (Fig. 7a–d). In line with their enhanced lytic ability, E7 TCR transduced T-cells were capable of producing overall greater quantities of IFN-γ and TNF-α than that of E6 TCR transduced T-cells (for IFN-γ against CASKi: 7785 pg/mL E7 vs. 1765 pg/mL for E6; for TNF-α: 7856 pg/mL E7 vs. 706 pg/mL E6). Together, these data indicate that this new manufacturing method results in highly functional T-cells that are capable of specifically recognizing and eliminating tumor cells expressing either the E6 or E7 target antigen in the context of HLA-A2.

## Discussion

The current work highlights the use of closed and semi-closed modular systems for clinical scale manufacturing of E6- and E7-specific TCR transduced T-cells. Manufacturing methods to generate TCR-transduced T-cells at some cell therapy centers generally utilize open lymphocyte enrichment, open 6-well plates for transduction, or open tissue culture flasks for expansion. Here, these procedures have been replaced with semi-automated closed lymphocyte enrichment, closed tissue culture bags, and closed G-REX containers, considerably limiting the potential for microbial contamination and increasing the consistency of the cultures. We show that using these methods, we are able to generate a large number of highly functional T-cells capable of recognizing target tumor cells. The process is reproducible and robust, and the performance of patient samples appears similar to those of healthy donor samples. This study used a single G-REX culture vessel to obtain approximately 20 × 10^9^ T-cells, but the cell yield can easily be increased further by using multiple vessels.

The manufacturing process was specifically designed so that it incorporated several distinct, but highly specialized modules so that it has a high degree of versatility and would be able to be performed at most standard cell processing facilities. First, automated enrichment of lymphocytes was achieved through the use of closed density gradient separation using the cobe2991 cell processor (Terumo, BCT). Next, after T-cells are activated and monocytes are removed, cells are transduced with retroviral vector and expanded for a period 7 days in closed culture bags. Part II of the manufacturing process incorporates a REP where transduced T-cells are stimulated with allogeneic irradiated feeder cells and anti-CD3 and allowed to further expand for 14 days within a closed GREX500. Upon cell harvest of one GREX500 using an automated closed GatheRex device, cells are in the range of 40 × 10^6^ cells/mL in a total of 500 mL. These cells are transferred to a LOVO cell wash/concentrate instrument (Fresenius Kabi) where they are washed with plasmalyte A containing HSA and resuspended at a final concentration of 10–330 × 10^6^ cells/mL for infusion (depending on dose level). One of the strengths of this manufacturing schema lies within its high degree of flexibility. This procedure would allow for the removal/insertion of other elements to easily fit the particular needs of multiple protocols, and we predict that this method will be effective for manufacturing other T-cell therapies. We previously found that G-REX flasks can be used for REP of tumor infiltrating lymphocytes (TIL), however, that method involved the use of smaller open system G-REX vessels and the open transfer of TIL at the time of harvest from the G-REX vessels [12]. The method described here involves the use of G-REX flasks with fivefold greater volume and when these larger vessels are used, media and cells can be added and removed without opening the system. Earlier renditions of this protocol and other protocols similar to this [20], which yielded ~ 20–30 × 10^9^ cells, required up to 40 T-175 flasks during the first 7 days of the REP. Each of these flasks required opening for media exchange at least once before they were finally transferred to cell culture bags for further expansion. The closing of the vessels and the reduction in total vessel number not only significantly reduce the volume of media and supplements required for manufacturing process, but also limit the chances of microbial contamination, and thus reduce the potential impact on patient well-being, cost and convenience. This system could also be used for manufacturing CAR T-cells, however, most CAR T-cell protocols do not require REP since a relatively small quantity of transduced T-cells are administered.

We find that this process results in a product that is very consistent. Although we observed slight differences in cell growth and transduction efficiency between E6 and E7 cultures, these same differences were not observed among the patient samples versus healthy donor within each of these groups and are most likely the result of the retroviral vector used during the manufacturing process.

Upon examining the kinetics of cell growth when using this procedure, we find that CD14+ monocytes are the first to disappear from the culture and are nearly completely eliminated by as early as day 2 of culture (Fig. 3b, d) due in large part to plastic adherence prior to transduction. This is followed by a disappearance of CD19+ B-cells, and finally CD56+ NK cells which persist in very small numbers outward to day 7 of culture, at which time point > 97% of the cells are CD3+ T-cells. Transduction efficiencies at day 7 are very high for both E6 transduced T-cells (> 68%) and E7 transduced T-cells (> 87%).

Resting T-cells exist in three general states, naïve (T_naive_), central memory (T_CM_), and effector memory (T_EM_) [21]. Most of the T-cells at the time of harvest using this procedure have the cellular phenotype of T_EM_ cells (> 90%), which is consistent with other products manufactured in a similar manner [22]. A potential limitation to this manufacturing process is that the current general consensus in the field is that a more naïve phenotype is associated with a more robust T-cell with the greater potential for long-term persistence. Clinical trials ongoing here at the NIH (NCT01087294) and by others are exploring this topic in more detail, and our group is currently testing a fully closed flow cytometer capable of sterilely sorting naive T-cells for these purposes (MACSQuant Tyto, Miltenyi).

Functional cell killing assays and cytokine release assays showed that the manufactured cells had a high degree of specificity for target antigen (Figs. 6, 7). In general, the main variations that were observed in these assays were again between E6 and E7 transduced cells rather than between healthy donor and patient samples within these groups, indicating potential differences in TCR affinity toward the target or other vector/construct related issues rather than differences stemming from the cell manufacturing process or patient variability.

## Conclusion

In summary, clinical manufacturing procedures for T-cell therapies that achieve a safe and consistent final product represents a major challenge in the field of cellular immunotherapy. Here, we addressed the issue of improving safe manufacturing procedures by introducing multiple semi-closed and closed-system modules aligned in tandem to generate E6- and E7-specific TCR modified T-cells. This process is currently being implemented here at the NIH Clinical Center for two ongoing trials (NCT03197025, NCT02858310). The modular nature of this approach allows for the highest degree of flexibility, which is of great benefit to most cell therapy manufacturing centers that must accommodate multiple phase I/II trials potentially using multiple cell types. Inherent inter-patient variability due to disease status and natural variation are among the factors that contribute the most to the challenge of obtaining a consistent final product. We are able to show that this manufacturing procedure results in a consistent final product and that there is little difference between products generated from healthy donors versus products generated from patients with HPV-associated cancers. Adaptation of these procedures at other phase I/II sites may help streamline the cell therapy manufacturing process, and allow for a quicker transition into later phase studies for successful products.
